# Effect of Heat Treatment Conditions on Mechanical Properties of Die-Casting Al–Si–Cu–xLa Alloys

**DOI:** 10.3390/ma18133046

**Published:** 2025-06-26

**Authors:** Kyeonghun Kim, Uro Heo, Younghun Bae, Seongtak Kim, NamHyun Kang, Haewoong Yang

**Affiliations:** 1 Pohang Institute of Materials Industry Advancement, Pohang 37666, Republic of Korea; kkh@pomia.or.kr (K.K.); uro@pomia.or.kr (U.H.); yhbae@pomia.or.kr (Y.B.); 2 Department of Materials Science and Engineering, Pusan National University, Busan 46241, Republic of Korea; 3 Korea Institute of Industrial Technology (KITECH), 47 Hojeo-ro, Wonju-si 26336, Republic of Korea; seongtak@kitech.re.kr

**Keywords:** aluminum alloys, lanthanum, heat treatment, mechanical properties

## Abstract

In this study, lanthanum (La), a rare earth element, was added at concentrations of 0.25 wt.%, 0.5 wt.%, and 0.75 wt.% to an Al–10%Si–2%Cu-based alloy prepared by die casting. The effects of solution and aging heat treatment conditions on the mechanical properties and corrosion resistance were investigated. Microstructural changes, hardness, and corrosion behavior were analyzed as functions of La content and heat treatment parameters. The optimal hardness was achieved at a solution treatment temperature of 500 °C or higher and an aging time of 2 h. In particular, the addition of 0.5 wt.% La led to significant refinement of the α-Al grains, enhancing hardness through the Hall–Petch strengthening mechanism. Furthermore, the combined effects of aging treatment and La addition promoted the formation of a fine, uniform microstructure and stable dispersion of precipitates, resulting in improved mechanical performance. Electrochemical polarization tests revealed that the alloy containing 0.5 wt.% La exhibited the best corrosion resistance. This enhancement was attributed to the formation of the LaCu_2_Al_4_Si intermetallic compound, which has a lower electrochemical potential than the Al_2_Cu phase, thereby reducing corrosion susceptibility within the microstructure.

## 1. Introduction

Aluminum alloys are representative lightweight materials extensively used across various industrial sectors, including aerospace, automotive, and defense. Among these, the Al–10%Si–2%Cu alloy is particularly utilized as a casting material due to its excellent fluidity and mechanical properties. Components manufactured by die casting, which is well suited for mass production and the fabrication of complex shapes, are increasingly being developed for diverse applications [1,2]. Typically, aluminum alloys exhibit improved properties through microstructural modifications achieved via heat treatment and alloying element additions [3,4,5]. However, in die-cast alloys, applying heat treatment is challenging because gas trapped during solidification can lead to porosity and subsequent gas release during heat exposure [6]. Despite this limitation, the demand for high-performance alloys is increasing with industrial advancements, prompting numerous studies focused on enhancing the properties of die-cast alloys through heat treatment [7,8,9]. According to R.N. Lumley, a composition similar to A380 (Al–8.5%Si–3.5%Cu) alloy demonstrated improved ductility and retained strength when subjected to T4 heat treatment at lower temperatures and shorter durations compared to conventional methods [10]. Celal Cingi investigated the effect of heat treatment on the thermal conductivity of Al–8%Si–3%Cu alloys and reported enhanced thermal conductivity, attributed to the migration of grain boundary precipitates into the aluminum matrix during treatment [11]. Qing Cai reported that, in Al–Si–Cu alloys, T6 heat treatment resulted in the dissolution of Q and Al_2_Cu phases into the α-Al matrix, yielding superior mechanical performance with tensile strength reaching 420 MPa and elongation exceeding 4% [12]. Although numerous studies have addressed heat treatment in die-cast alloys, limited research has examined the simultaneous effects of alloying element addition and heat treatment on performance enhancement. This study aims to identify the optimal conditions for improving the mechanical properties of die-cast Al–10%Si–2%Cu–xLa alloys through controlled heat treatment.

## 2. Materials and Methods

Al–10%Si–2%Cu, Al–20%La, and Al–10%Sr master alloys were used as the base materials, and Al–10%Si–2%Cu–xLa alloys were fabricated via die casting. The die-casting process was conducted under the following conditions: a clamping force of 350 tons, a melt temperature of 700 °C, and a mold temperature of 300 °C. The La content was varied at 0, 0.25 wt.%, 0.5 wt.%, and 0.75 wt.%. The alloy compositions, as measured by wavelength-dispersive X-ray fluorescence (WD-XRF; M4 Tornado, Bruker, Billerica, MA, USA), are presented in Table 1. To eliminate the influence of surface impurities, central specimens were extracted and used for all characterization analyses. Heat treatment involved solutionizing at temperatures ranging from 440 °C to 540 °C in 20 °C intervals, followed by water quenching and subsequent artificial aging at 200 °C for durations from 0 to 12 h in 2 h increments [13]. The final testing conditions were selected based on optimal parameters identified in previous studies. Specimens were ground using 120–2400 grit SiC abrasive papers and then polished using an oxide polishing suspension (OP-S). The α-Al microstructure was examined using an optical microscope, and the area fraction of α-Al was quantified in five randomly selected regions using ImageJ software. Precipitate analysis was conducted using scanning electron microscopy (SEM, SU5000, HITACHI, Tokyo, Japan) and energy-dispersive X-ray spectroscopy (EDS) under the conditions of 15 kV accelerating voltage and 10 mm working distance. Additional microstructural characterization was carried out by electron backscatter diffraction (EBSD) with an accelerating voltage of 20 kV and a step size of 0.1 µm, and validated with X-ray diffraction (XRD, JP/Max-3A, Rigaku, Tokyo, Japan) using Cu Kα_1_ radiation (λ = 1.540593 Å) at 45 kV and 200 mA. The scan range for XRD analysis was set from 5° to 100° in the Theta/2-Theta configuration. Mechanical properties were evaluated by microhardness testing (HV; Mitutoyo HM220D, Kawasaki-shi, Kanagawa, Japan) and tensile testing (UTS; MTS Landmark, San Diego, CA, USA). The microhardness test was conducted under a load of HV 0.1, and the tensile test specimens had a width of 6 mm and a gauge length of 25 mm. Corrosion behavior was assessed via potentiodynamic polarization using a three-electrode electrochemical cell (Autolab PGSTAT302N, Herisau, Switzerland) with an exposed working electrode area of 50 mm^2^. A platinum plate and an Ag/AgCl electrode served as the counter and reference electrodes, respectively. Prior to measurement, the electrolyte solution was purged with nitrogen gas for 15 min to eliminate dissolved oxygen. Corrosion data were analyzed using Autolab NOVA 2.1 software.

## 3. Results

**Figure 1:**
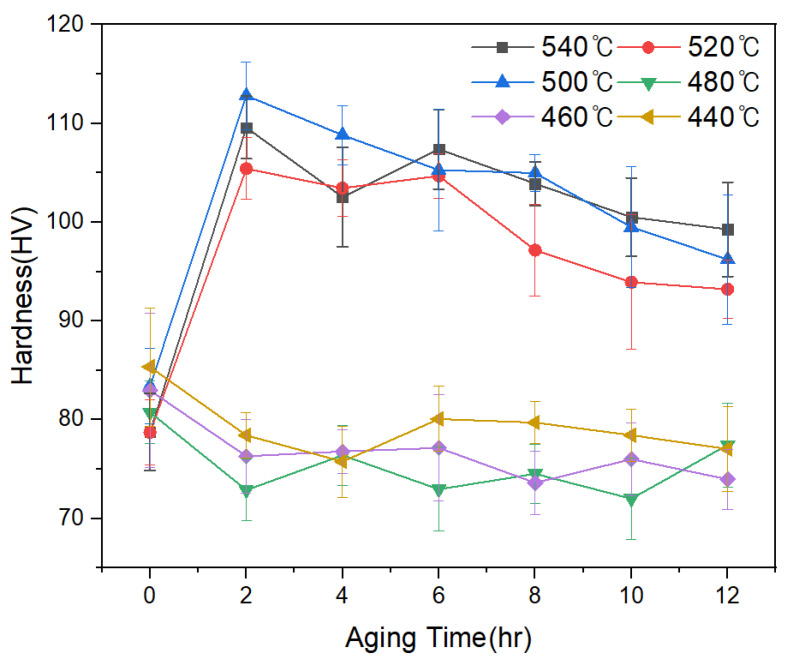
Average micro-Vickers hardness values of ASDC–La 0 wt.% alloy under various solution treatment and aging conditions.

Figure 1 presents the micro-Vickers hardness values of the die-cast Al–Si–Cu alloy as a function of solution treatment temperature and aging time. The as-cast samples exhibited an average hardness of approximately 95 HV. The samples subjected only to solution treatment exhibited hardness values ranging from the upper 70s to mid-80s HV, with minimal variation. In contrast, specimens that underwent aging heat treatment after solutionizing at temperatures between 500 °C and 540 °C displayed significantly higher hardness. The maximum hardness values were recorded at 500 °C (112 HV), 520 °C (105 HV), and 540 °C (109 HV) after 2 h of aging. These values represent an increase of approximately 10% in hardness compared to the as-cast condition, under the most favorable treatment conditions. However, for solution treatment temperatures below 500 °C (i.e., 440–480 °C), aging led to a reduction in hardness, indicating a negligible aging response. Therefore, the most favorable conditions for achieving enhanced hardness were identified as solution treatment at or above 500 °C, followed by aging for 2 h.

### 3.1. Results of Microstructure Analysis According to Heat Treatment and La Addition

**Figure 2:**
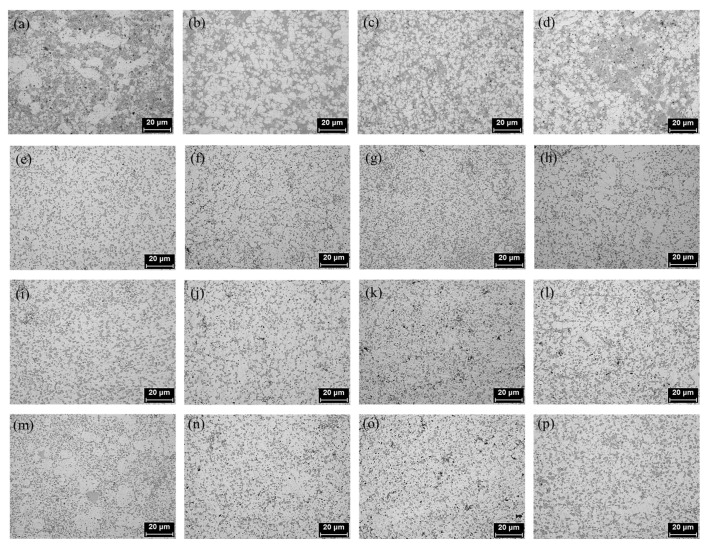
Optical micrographs of Al–Si–Cu–xLa alloys; (**a**–**d**) aging at 0 h of Al-Si-Cu-xLa (0, 0.25, 0.5, 0.75), (**e**–**h**) aging at 2 h of Al-Si-Cu-xLa (0, 0.25, 0.5, 0.75), (**i**–**l**) aging at 6 h of Al-Si-Cu-xLa (0, 0.25, 0.5, 0.75), and (**m**–**p**) aging at 12 h of Al-Si-Cu-xLa (0, 0.25, 0.5, 0.75), respectively.

Figure 2 shows the optical microstructures of the alloys with varying La content after solution treatment followed by aging at 0 h, 2 h, 6 h, and 12 h. Typically, Al–Si alloys solidify with dendritic structures; however, in die-cast alloys, dendritic morphology is generally absent due to the high cooling rate during casting and the subsequent effects of heat treatment [13]. In all specimens, equiaxed grains composed of α-Al and eutectic Si phases were observed. Spheroidization of eutectic Si was evident after aging treatment, indicating microstructural refinement under the applied thermal conditions (Figure 2a–d). With aging times beyond 6 h, coarsening of α-Al grains was observed, suggesting grain growth at extended durations [14]. These microstructural changes indicate that the aging response is closely tied to heat treatment conditions and La content, influencing grain refinement and phase distribution.

**Figure 3:**
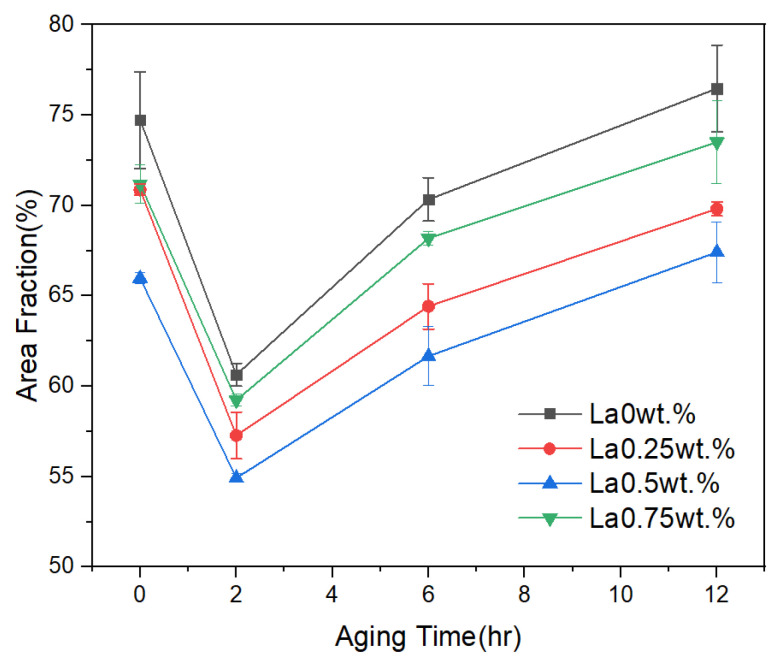
Area fraction analysis of α-Al grains by La content after solution heat treatment and aging for 0 h, 2 h, 6 h, and 12 h.

**Figure 4:**
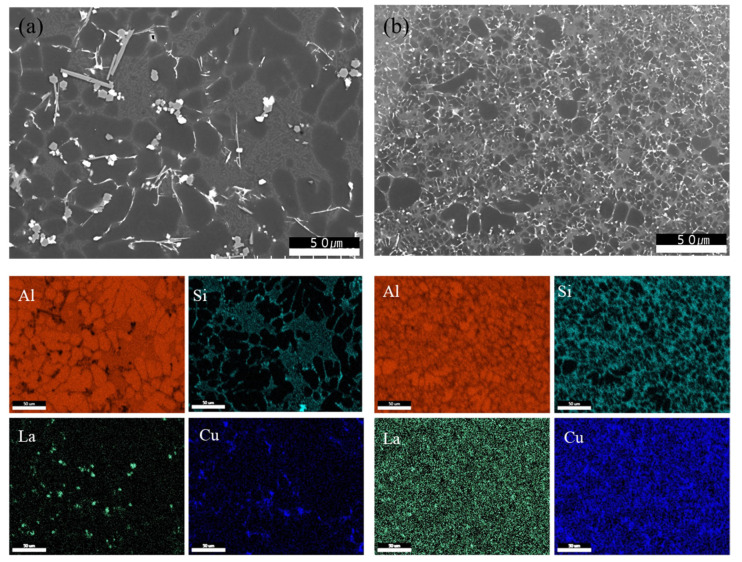
Electron microscopy and EDS mapping of the La0.5 wt.% alloy: (**a**) without aging heat treatment; (**b**) after 2 h of aging heat treatment.

Structural variations were observed depending on the presence and content of La and the application of heat treatment. The refinement effect on α-Al grains was quantitatively assessed using the Otsu thresholding method in ImageJ. The results are summarized in Figure 3 and Table 2. For specimens subjected only to solution treatment, the average α-Al area fractions across five measurements were 74.73%, 70.86%, 65.97%, and 71.18% for La contents of 0, 0.25, 0.5, and 0.75 wt.%, respectively. After 2 h of aging heat treatment, the α-Al fractions decreased to 60.63%, 57.27%, 54.92%, and 57.22%, indicating a refinement of the α-Al phase. Notably, a reduction of approximately 17% was observed at 0.5 wt.% La. These microstructural changes can be attributed to the spheroidization of eutectic Si during aging. In the as-cast condition, the rapid cooling rate inhibited diffusion and phase growth, thereby preventing spheroidization. However, aging provided sufficient thermal exposure to promote the diffusion of Si atoms along the Al–Si interface, leading to the gradual transformation of eutectic Si into a spheroidized morphology [15]. Coarsening of eutectic Si was observed after aging durations exceeding 6 h, which can be explained by Ostwald ripening. Over time, smaller Si particles, possessing higher surface energy and greater thermodynamic instability, tend to dissolve into the matrix. In contrast, larger Si particles are energetically more stable due to their lower surface-to-volume energy ratio. Dissolved Si atoms diffuse through the Al matrix and redeposit on the surfaces of the coarser Si particles. As this process repeats, smaller precipitates dissolve, larger ones grow, the total number of precipitates decreases, and the average particle size increases. Figure 4 presents electron microscopy and EDS mapping images of the alloy containing 0.5 wt.% La. In the sample without aging, the microstructure remains relatively unrefined, whereas after 2 h of aging heat treatment, a fine and uniform distribution of Al and Si phases is clearly visible, confirming the refining effect of La in conjunction with thermal exposure.

In addition, relatively coarse α-Al grains were observed in the alloy without La addition (Figure 2a–d) under identical heat treatment conditions. In contrast, the La-added alloys (all samples excluding Figure 2a–d) exhibited visibly refined α-Al grains. Among these, the alloy containing 0.5 wt.% La showed the finest grain structure, while the alloy with 0.75 wt.% La exhibited relatively coarser grains. Nevertheless, all La-added alloys demonstrated a refined structure compared to the La-free counterpart, confirming that La addition promotes α-Al grain refinement. This observation aligns with findings from our previous study involving gravity casting of Al–Si–Cu alloys with La additions. The grain refinement mechanism is attributed to the segregation of La atoms at the solid–liquid interface during aluminum solidification. This segregation induces a compositional subcooling effect due to the disparity between the actual cooling rate and the equilibrium solidification temperature in the presence of La. This subcooling facilitates the formation of La-rich intermetallic compounds, which act to inhibit α-Al grain growth and promote refinement [16,17]. This effect is further supported by the growth restriction factor (GRF), which quantitatively expresses the influence of solute elements on grain refinement. The GRF for La is 2.0279, significantly higher than the value for Ce (0.186), another rare earth element with similar characteristics [18]. A higher GRF indicates a stronger solute-induced suppression of grain growth, as it promotes rapid local subcooling around the nucleation site. This accelerates the solidification of the surrounding liquid metal, enhancing stable nucleation and grain refinement [19]. Thus, the grain-refining effect of La observed in die-cast Al–Si–Cu alloys is consistent with theoretical expectations and previously reported experimental results.

### 3.2. Analysis of Mechanical Properties by Heat Treatment and La Addition

**Figure 5:**
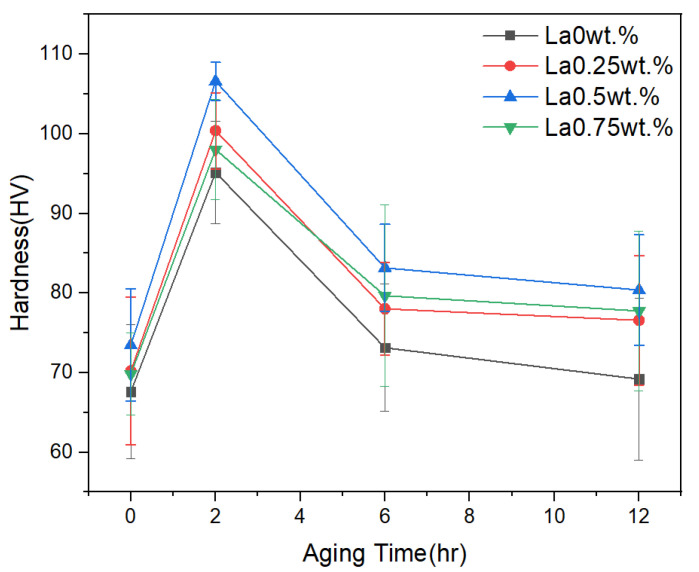
Average hardness results of Al–Si–Cu–xLa alloys after solution heat treatment and aging for 0 h, 2 h, 6 h, and 12 h. Hardness variation with La content.

Figure 5 presents the mechanical property results as a function of La content under aging durations of 0 h, 2 h, 6 h, and 12 h following solution treatment. The average hardness values without aging were 67.61 HV, 70.21 HV, 73.47 HV, and 69.82 HV, corresponding to increasing La content. After 2 h of aging, the hardness increased significantly, reaching 95.15 HV, 100.41 HV, 106.60 HV, and 98.04 HV, respectively. These values represent an increase of approximately 41% in hardness with La addition compared to the unaged, La-free alloy. However, with prolonged aging (6 h and 12 h), the hardness declined across all compositions. At 12 h, the hardness of the La-free alloy decreased by approximately 28% compared to the 2 h condition. This trend was consistent regardless of La content, indicating that hardness variation is primarily influenced by the formation and coarsening of precipitates within the supersaturated solid solution formed during solution treatment. Under the 0 h condition (solution treatment only), limited precipitation strengthening resulted in low hardness. After 2 h of aging, fine precipitates formed and effectively hindered dislocation movement, significantly enhancing hardness. However, with extended aging, these precipitates coarsened, reducing their ability to impede dislocations and thus diminishing the strengthening effect. Among the La-containing alloys, the highest hardness was observed at 0.5 wt.% La. Although the hardness slightly decreased at 0.75 wt.% La, it remained higher than that of the 0 wt.% La alloy.

(1) σy=σ₀+k_y×d^(−1/2)

where σy is the yield strength, *σ*_0_ is the lattice friction stress (a constant independent of grain size), k_y is the Hall-Petch constant (material-specific constant), and *d* is the average grain size [20].

According to this equation, a reduction in grain size increases the grain boundary area per unit volume, enhancing strength by impeding dislocation movement. The highest hardness at 0.5 wt.% La is attributed to optimal grain refinement under this composition. However, at 0.75 wt.% La, the nucleation-promoting effect was diminished due to saturation of La at the solid–liquid interface, reducing its ability to suppress grain growth. This led to grain coarsening and a subsequent reduction in hardness.

**Figure 6:**
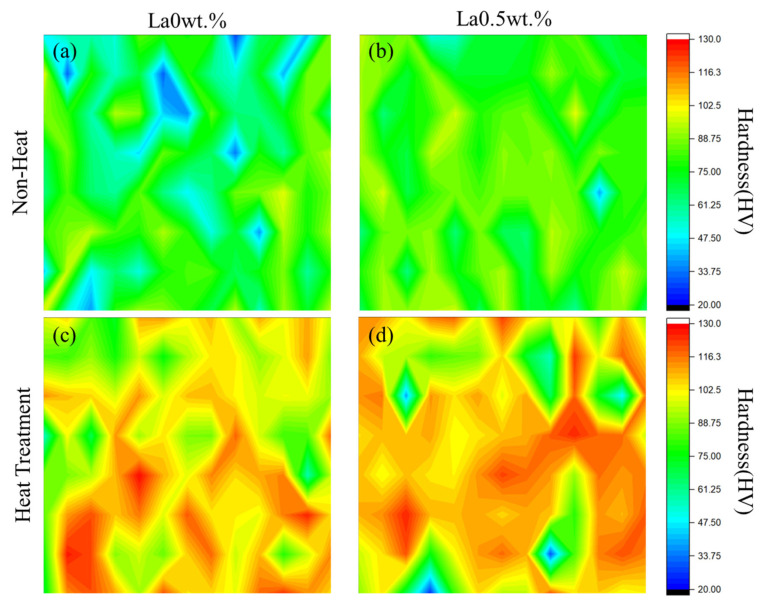
Hardness mapping of alloys with and without La addition under 0 h and 2 h aging conditions. (**a**) La0wt.% alloy before aging, (**b**) La0.5wt.% alloy before aging, (**c**) La0wt.% alloy after aging, and (**d**) La0.5wt.% alloy after aging.

In addition, the effect of La on grain uniformity was evaluated through micro-Vickers hardness mapping, as shown in Figure 6. For the La 0 wt.%, 0.25 wt.%, and 0.5 wt.% alloys under 0 h and 2 h aging conditions, hardness was measured at 100 points over a 1 cm × 1 cm area. In the absence of aging, the hardness maps revealed broader variation and lower average values (dominantly green and blue regions), indicating a heterogeneous microstructure. After 2 h of aging, particularly in La-added samples, an increase in overall hardness and a more uniform distribution (dominantly yellow and red regions) were observed. These results suggest that La addition not only enhances hardness through grain refinement and precipitation hardening but also contributes to microstructural homogeneity during heat treatment by promoting uniform grain growth and element diffusion.

### 3.3. Analysis of Corrosion Resistance Characteristics According to Heat Treatment and La Addition

**Figure 7:**
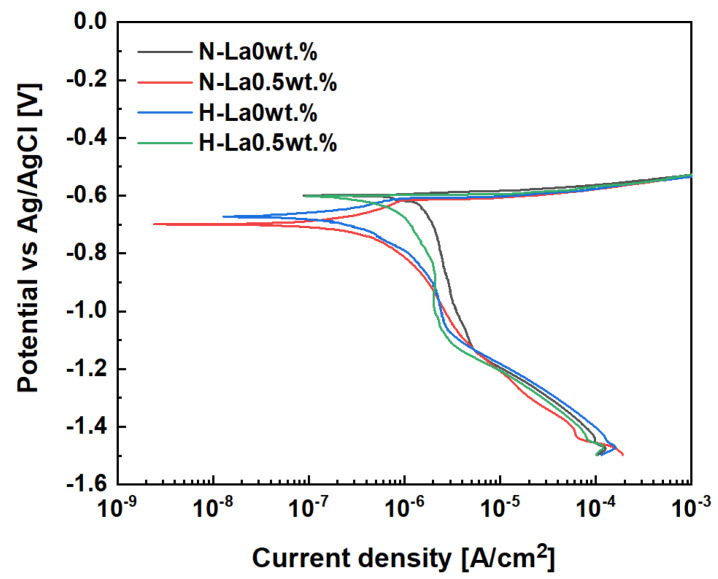
Tafel polarization curves of Al–Si–Cu–xLa alloys in 3.5 wt.% NaCl solution.

The corrosion behavior of Al–Si–Cu–xLa alloys was evaluated using Tafel polarization curves, as shown in Figure 7. While the anodic regions displayed nearly identical behavior across all samples, a distinct shift toward the left was observed in the cathodic region with La addition and heat treatment. This shift indicates a reduction in cathodic reaction kinetics and implies that both La addition and heat treatment effectively suppress the corrosion rate. Table 3 presents the corrosion resistance values of each alloy as a function of La content and aging heat treatment duration. In electrochemical testing, higher Ecorr values or lower Icorr values indicate improved corrosion resistance [21]. The Ecorr and Icorr values were obtained from the Tafel polarization curves. For Ecorr (corrosion potential), no significant differences were observed in the untreated samples, and the heat-treated alloys showed only a minor variation of 0.021 V—within the standard error range—indicating a negligible effect. In contrast, Icorr (corrosion current density) varied significantly depending on the presence of heat treatment and La addition. The untreated La-free specimen (N-La 0 wt.%) recorded an Icorr of 1.27 × 10^−6^ A/cm^2^, whereas the specimen with 0.5 wt.% La (N-La 0.5 wt.%) showed a reduction of approximately 47% to 6.65 × 10^−7^ A/cm^2^. Among the heat-treated specimens, La addition reduced Icorr by 5%, and the difference between N-La 0 wt.% and H-La 0.5 wt.% reached 82%, indicating a substantial improvement in corrosion resistance due to the combined effects of La addition and heat treatment.

In addition, polarization resistance was calculated using the Stern–Geary equation to evaluate the corrosion resistance characteristics based on the Tafel data. In Equation (2), *β_a_* and *β_c_* represent the Tafel slopes of the anodic and cathodic reactions, respectively, and *I_corr_* denotes the corrosion current density [22].

(2) $$R_{p}=\frac{\beta_{a}\times \;\beta_{c}}{2.3\;I_{corr}\;(\beta_{a}\;+\beta_{c})}$$

The corrosion resistance, calculated using Equation (2), showed a significant improvement of approximately 570% for the H-La 0.5 wt.% alloy, which exhibited a polarization resistance of 6.42 × 10^4^ Ω·cm^2^, compared to 9.29 × 10^3^ Ω·cm^2^ for the N-La 0 wt.% alloy. Even after aging heat treatment, La addition improved corrosion resistance by approximately 60%. To further evaluate the corrosion performance, the corrosion rate (mm/y), reflecting material loss, was calculated using Equation (3). In this equation, Icorr is the corrosion current density (A/cm^2^), *K* is a constant (3272 mm·A^−1^·cm·year^−1^, per ASTM G102 [23]), *EW* is the equivalent weight (g/eq), ρ is the specimen density (g/cm^3^), and A is the surface area (cm^2^). The calculated annual mass loss rate for the H-La 0.5 wt.% alloy was the lowest at 0.00038 mm/y, indicating superior corrosion resistance among the tested conditions [24].

(3) $$\mathrm{Corrosion}\;\mathrm{rate}=\frac{I_{corr}\;K\;EW}{\rho A}$$

**Figure 8:**
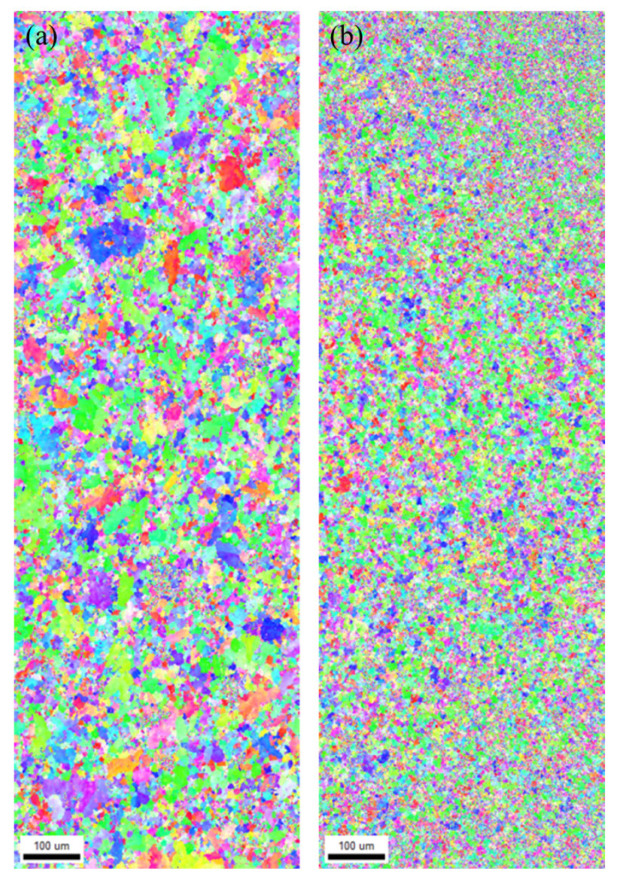
EBSD analysis of the La0.5 wt.% alloy: (**a**) before and (**b**) after 2 h of aging heat treatment.

To date, the mechanisms underlying corrosion resistance improvements due to microstructural changes have not been fully elucidated [18]. However, this study identifies two primary factors contributing to the enhanced corrosion resistance. The first is microstructural refinement. Figure 8 presents EBSD results of the 0.5 wt.% La alloy before and after 2 h of aging. The inverse pole figure map (Figure 8b) of the aged sample displays a significantly refined α-Al grain structure. According to prior studies, fine-grained aluminum microstructures facilitate rapid formation of uniform and adherent natural oxide layers, thereby enhancing corrosion resistance [25,26]. These observations support the conclusion that corrosion resistance in this study is closely associated with grain refinement induced by heat treatment.

**Figure 9:**
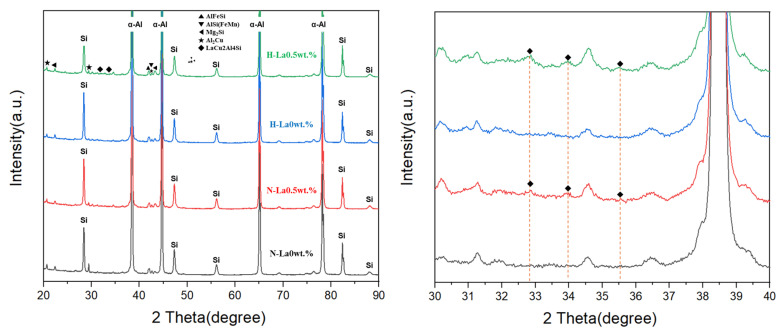
XRD patterns of the La 0.5 wt.% alloy in solutionizing and aged conditions.

The second contributing factor is the formation of specific intermetallic precipitates. As shown in the XRD patterns in Figure 9, the LaCu_2_Al_4_Si phase was identified in the La-added Al–Si–Cu alloys. This phase is known to possess a lower electrochemical potential than the Al_2_Cu phase, which is a major cathodic constituent in corrosion processes of Al–Si–Cu systems [27]. The substitution of Al_2_Cu by LaCu_2_Al_4_Si, therefore, contributes to lowering the corrosion driving force. Consequently, the improved corrosion resistance observed in the La-added alloys can be attributed to the formation of this less noble intermetallic compound [28].

**Figure 10:**
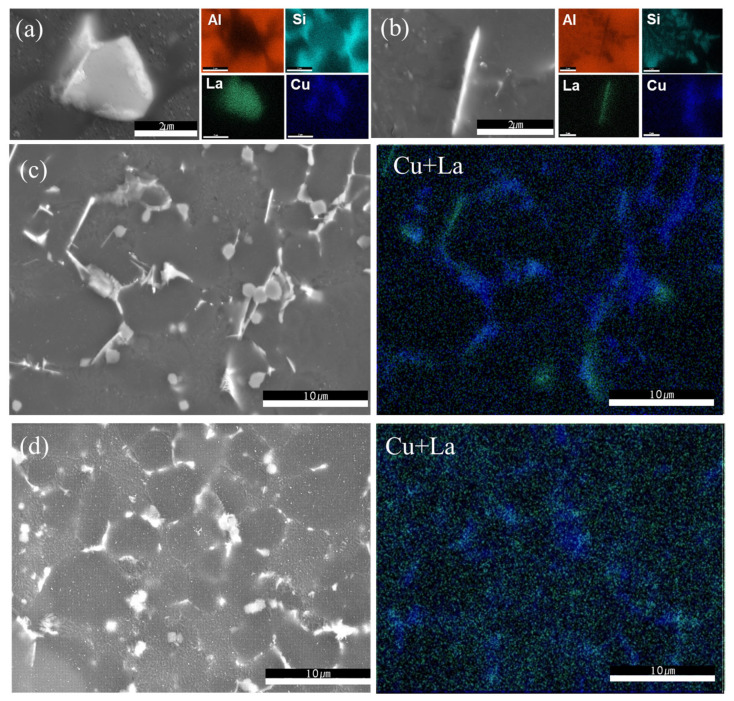
EDS analysis of LaCu_2_Al_4_Si phase morphology and Cu/La distribution in the La0.5 wt.% alloy: (**a**) La phase before aging, (**b**) La phase after aging, (**c**) microstructure before aging, and (**d**) microstructure after aging.

Confirming the formation of LaCu_2_Al_4_Si.The presence and morphology of the LaCu_2_Al_4_Si phase were confirmed via electron microscopy and EDS analysis, as shown in Figure 10a,b. These intermetallics were observed in both needle-like and polygonal forms, regardless of the aging heat treatment condition. The needle-like morphology is known to induce localized stress concentration, which negatively impacts corrosion resistance. In contrast, the polygonal form, due to its lower geometric stress concentration, is associated with reduced corrosion sensitivity [29,30,31]. In the as-cast alloys, precipitates appeared relatively coarse and were primarily concentrated along grain boundaries. After aging treatment, the precipitates exhibited a finer, more uniform distribution along the grain boundaries, with minimal aggregation [32]. This uniformity is clearly supported by the overlapped EDS maps of Cu and La in Figure 10c,d, which reveal evenly dispersed secondary phase particles formed from the supersaturated solid solution during aging. These fine precipitates also contribute to microstructural stabilization through a Zener drag effect, which arises during heat treatment. This phenomenon resists grain boundary migration by exerting pinning pressure, particularly during boundary movement driven by curvature minimization [33]. The presence of finely distributed precipitates restricts this migration, suppressing grain growth and leading to the formation of refined α-Al grains and a homogeneous microstructure [34,35,36].

## 4. Conclusions

The optimal heat treatment condition for maximizing the hardness of the Al–Si–Cu alloy was identified as solution treatment at 500 °C or higher, followed by 2 h of aging. This aging treatment facilitated the spheroidization of eutectic Si and promoted the grain growth of α-Al. However, when the aging time exceeded 6 h, Ostwald ripening occurred, resulting in the coarsening of the Si phases. The addition of La proved beneficial in refining the α-Al grains by segregating at the solid–liquid interface during solidification, thereby inducing a compositional undercooling effect. This grain refinement led to increased hardness in accordance with the Hall–Petch relationship, with the 0.5 wt.% La-added alloy achieving the highest hardness value of 106.6 HV. Furthermore, the H-La 0.5 wt.% alloy (containing 0.5 wt.% La and aged for 2 h) exhibited the best corrosion resistance, with a polarization resistance of 6.42 × 10^4^ Ω·cm^2^ and a corrosion current density of 2.20 × 10^−7^ A/cm^2^. This enhancement is attributed to the presence of fine α-Al grains and uniformly distributed precipitates, which promote the formation of a dense and protective oxide film. These results collectively demonstrate that the combined application of La addition and optimized heat treatment (solutionizing at 500 °C followed by 2 h of aging) significantly improves both the mechanical properties and corrosion resistance of Al–Si–Cu alloys.

## Figures and Tables

**Table 1:** Chemical composition of ASCD–xLa alloys (wt.%).

Alloys	Si	Mg	Ti	Mn	Fe	Cu	Zn	Sr	La	Al
ASCD-0	9.18	0.25	0.03	0.22	0.69	1.50	0.94	0.02	-	Bal.
ASCD-0.25	9.44	0.28	0.08	0.21	1.06	1.50	1.07	0.02	0.28	
ASCD-0.5	8.96	0.24	0.02	0.21	0.66	1.42	0.90	0.02	0.45	
ASCD-0.75	8.48	0.26	0.02	0.22	0.65	1.41	0.89	0.02	0.66	

**Table 2:** Area fraction (%) of α-Al phase by La content and aging time.

Alloys Aging Time (h)	La0wt.%		La0.25wt.%		La0.5wt.%		La0.75wt.%	
	Area Fraction (%)	SD.	Area Fraction (%)	SD.	Area Fraction (%)	SD.	Area Fraction (%)	SD.
0	74.73	2.67	70.86	0.30	65.97	0.31	71.18	1.06
2	60.63	0.62	57.27	1.29	54.92	0.23	57.22	0.32
6	70.33	1.20	67.41	1.24	61.66	1.63	68.18	0.37
12	76.47	2.40	71.81	0.38	67.41	1.66	73.51	2.30

**Table 3:** Electrochemical parameters obtained from Tafel polarization curves of Al–Si–Cu–xLa alloys in 3.5 wt.% NaCl.

Alloys	I_corr_ (A/cm^2^)	E_corr_ (V)	Corrosion Rate (mmy)	Βanodic (V/Decade)	βcathodic (V/Decade)	Rp (Ω·cm^2^)	I_corr_ (A/cm^2^)
N-La0wt.%	1.27 × 10^−6^	0.603	1.38 × 10^−2^	0.0292	0.398	9.29 × 10^3^	1.27 × 10^−6^
N-La0.5wt.%	6.65 × 10^−7^	0.603	7.25 × 10^−3^	0.0409	0.366	2.40 × 10^4^	6.65 × 10^−7^
H-La0wt.%	4.34 × 10^−7^	0.673	4.74 × 10^−3^	0.0354	0.312	3.91 × 10^4^	4.34 × 10^−7^
H-La0.5wt.%	2.20 × 10^−7^	0.692	2.41 × 10^−3^	0.0353	0.408	6.42 × 10^4^	2.20 × 10^−7^

## Data Availability

The original contributions presented in this study are included in the article. Further inquiries can be directed to the corresponding authors.
